# Molecular Iodine Exhibited Differential Antiproliferative Actions in Progenitor and Stem Populations from Chemoresistant Cancer Cells

**DOI:** 10.3390/ijms26094020

**Published:** 2025-04-24

**Authors:** Irasema Mendieta, Jazmin Leon-Pichardo, Gustavo Orizaga-Osti, Edgar R. Juvera-Avalos, Uriel Rangel-Chavez, Evangelina Delgado-Gonzalez, Brenda Anguiano, Carmen Aceves

**Affiliations:** Instituto de Neurobiología, Universidad Nacional Autonoma de Mexico, Queretaro 76230, Mexico; aliciairasema@hotmail.com (I.M.); jazminpichardo.8@gmail.com (J.L.-P.); orizaga.osti@gmail.com (G.O.-O.); rodrigojuvera@gmail.com (E.R.J.-A.); edelgado@comunidad.unam.mx (E.D.-G.); anguianoo@unam.mx (B.A.)

**Keywords:** cancer stem cells, neuroblastoma, molecular iodine, zebrafish xenograft

## Abstract

Cancer stem cells (CSCs) are described as a subpopulation of cells with capabilities of self-renewal, chemoresistance, and invasiveness. CSCs reside in tumor niches and can be studied in vitro through their enrichment in spheroids (Stem). Molecular iodine (I_2_) induces apoptosis and differentiation in various cancer cells. I_2_ can activate peroxisome proliferator-activated receptors type gamma (PPARγ), and its pathways are associated with its oxidant/antioxidant capacity. This work aimed to compare the effect of I_2_ supplementation in progenitor and CSC populations with low (MCF-7 and S-K-NAS) and high invasiveness (MDA-MB231 and SK-N-BE2) in mammary and neuroblastoma (NB) cell lines. Results showed that the CSC population enriched by the spheroid culture overexpressed stem messengers CD44, SOX2, and NMYC and exhibited the highest mitochondrial metabolism (membrane mitochondrial potential and O_2_^−^). The presence of I_2_ increases PPARγ expression and induces apoptosis through the Bax/Bcl2 index in all populations but silences NMYC expression and reduces mitochondrial metabolism in Stem NB. I_2_ also enhances the expression of nuclear erythroid factor 2 (Nrf2) in all populations, but the target antioxidant superoxide dismutase 2 (SOD2) is only elevated in progenitor cells. In contrast, the mitophagy inductors PTEN-induced putative kinase 1 (Pink1) and microtubule-associated protein1 light chain3 alpha (LC3) were overexpressed in Stem populations. I_2_-preselected SK-N-BE2 populations exhibited minor implantation and invasion capacities in the in vivo zebrafish model. These data indicate that I_2_ interferes with viability, implantation, and invasion capacity in all cell lines, but the molecular mechanisms vary depending on the progenitor or Stem condition.

## 1. Introduction

Cancer is the second leading cause of death worldwide. Successful therapeutic approaches have been developed that have significantly reduced mortality rates; however, challenges persist for so-called high-grade cancers that present characteristics such as chemoresistance and high invasiveness. Two examples of these challenges are triple-negative breast cancer (TNBC) and neuroblastoma with NMYC gene overexpression [1].

Breast cancer (BC) is the most common cancer in women. Its molecular classification includes four main subtypes: luminal A and B, tumors with human epidermal growth factor receptor 2 (HER2) overexpression, and TNBC. The luminal A subtype is the most prevalent and is characterized by the expression of estrogen receptors (ER) and progesterone receptors (PR). It is also considered the least aggressive. The luminal B subtype is characterized by the expression of ER and PR, along with HER2 overexpression and a high rate of cell proliferation. HER2-positive tumors are negative for hormone receptors and overexpress the HER2 protein, which is highly associated with gene overamplification. Finally, the TNBC subtype is characterized by the absence of ER, PR, and HER2 expression. It accounts for 15% to 20% of new cases and has the worst prognosis, as it is associated with aggressive histological features and poor patient survival, and is generally chemoresistant [2].

Neuroblastoma (NB) is a neoplasm of the sympathetic nervous system derived from neural crest cells and is the second most common extracranial tumor in childhood. NB is characterized by a heterogeneous clinical presentation and evolution, ranging from spontaneously regressing neoplasms to highly metastatic and chemoresistant tumors (high-grade NB). Tumors with a good prognosis respond to chemotherapy and are sensitive to differentiation factors such as retinoic acid. In contrast, the high-grade NB are resistant to conventional chemotherapy treatments and overexpress the MYCN gene [3]. The human MYC gene (encoding the CMYC protein) was identified as a cellular homolog of an avian retroviral transforming gene, v-myc. MYC family is composed of three closely related genes, MYC (encoding Cellular-MYC), MYCN (encoding Neural-MYC), and MYCL (encoding Lung-MYC), identified in epithelial cells, neuroblastoma, and small cell lung cancer, respectively. NMYC is overexpressed in both nervous system tumors (e.g., neuroblastoma, medulloblastoma, retinoblastoma, astrocytoma, and glioblastoma multiforme) and non-neuronal tumors (neuroendocrine prostate cancer, hematologic malignancies, and pancreatic tumors, among others). The persistent overexpression of NMYC in quiescent, self-renewing stem cells suggests a role for this gene in maintaining stem cell-like properties (e.g., self-renewal, chemoresistance, etc.), suggesting its potential as a determinant of high-grade neuroblastoma [4]. Ectopic expression of NMYC in zebrafish neural crest also induces neuroblastoma, demonstrating that the neuroblastoma-inducing potential of NMYC is conserved across species [5].

Cancer stem cells (CSCs) are a subpopulation of cancer cells with self-renewal capacity, tumorigenicity, chemoresistance, and invasiveness that reside in specialized tumor niches and may be responsible for therapeutic resistance [6]. CSCs can be studied in vitro through their enrichment in spheroids in low-adherence culture plates without differentiating factors [7]. TNBC and NMYC-positive NB exhibit chemoresistant and highly metastatic rates, and CSCs have been described in both primary tissue and immortalized cell lines of these cancer types [1,7].

Molecular iodine (I_2_) exerts antiproliferative and apoptotic effects in several cancer cells [8,9,10,11]. These effects are related to its oxidant/antioxidant capacity, which disrupts the mitochondrial membrane potential (MmpΨ), leading to the release of apoptosis-inducing factor [12], or through the activation of peroxisome proliferator-activated receptors type gamma (PPARγ) [13]. I_2_ supplementation in vitro studies reduced the gene expression associated with CSC patterns and invasion potentiality [14,15]. I_2_ supplementation in vivo models with conventional therapies led to a 4- to 10-fold decrease in drug concentration while maintaining the antineoplastic effect and significantly attenuating side effects such as body-weight loss, diarrhea, and hemorrhagic cystitis [16,17,18]. This work aims to analyze the impact of I_2_ on the viability and mitochondrial functional state of progenitor and Stem populations in mammary (MCF-7 and MDA-MB231) and NB (SK-NAS and SK-N-BE2) cell lines. Additionally, this work describes the implantation and invasion capabilities of an I_2_-preselected NB cell population in the in vivo zebrafish model.

## 2. Results

### 2.1. I_2_ Supplementation Diminishes the Cell Line’s Viability

**Figure 1:**
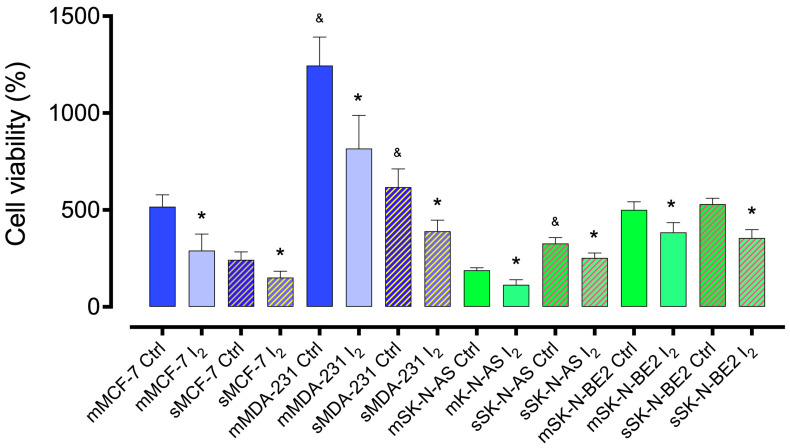
Cell viability. Effect of 200 µM I_2_ on the viability of mammary (blue) and neuroblastoma (green) cell line progenitors (monolayer; m) or Stem (spheroids; s) after 96 h of treatment. All experiments were carried out using the trypan blue dye exclusion assay. Data represent three independent experiments conducted in duplicate and are expressed as the mean ± SD. Statistical difference (*p* < 0.05) intragroup (*, Student’s *t*-test) and intergroup (&, one-way ANOVA/Tukey’s), respectively.

Cell viability was analyzed by trypan dye exclusion after 96 h of culture. Figure 1 shows that progenitor (monolayer; m) cells exhibited a lower proliferation rate in the less aggressive cell lines (MCF-7 and SK-NAS) than the most aggressive ones (MDA-MB231, 2.4 times; SK-N-BE2, 2.2 times). Interestingly, the Stem culture (spheroid; s) exhibited a lower proliferation rate than progenitor cells in the mammary cell lines; however, in the NB cell line, the proliferation rates of both populations were similar or elevated, corroborating the high aggressiveness of this cell line. I_2_ supplementation inhibited cell viability in all cell lines, regardless of their culture conditions.

### 2.2. I_2_ Supplementation Diminishes the Formation of Spheroid and Stem Marker Expression

**Figure 2:**
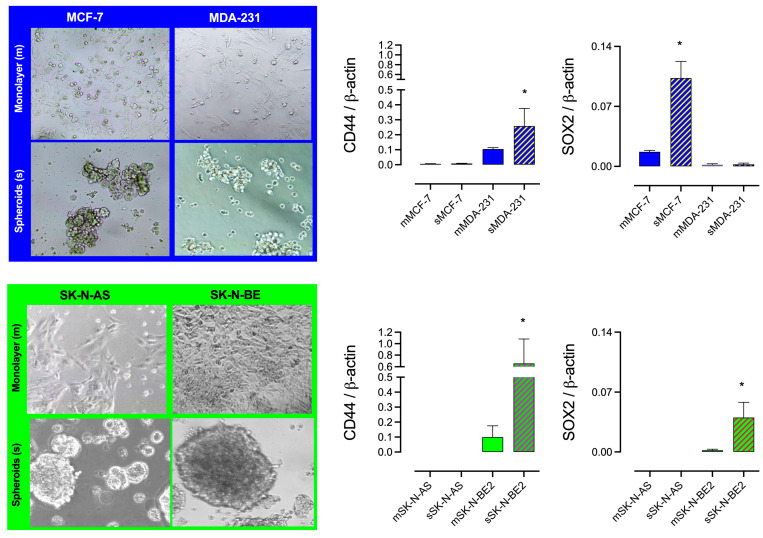
CSC enrichment. Representative micrographs (10×) of spheroid formation and stem marker (CD44 and SOX2) expression (RT-qPCR) in monolayer (m) or spheroid (s) cultures in mammary (blue) and neuroblastoma (green) cell lines. All experiments were carried out for 96 h (mammary) and 120 h (neuroblastoma). Data represent three independent experiments conducted in duplicate and are expressed as the mean ± SD. * indicates statistical differences between groups (Student’s *t*-test; *p* < 0.05).

Figure 2 presents representative micrographs of progenitor (monolayer; m) and Stem (spheroid; s) cultures (96 h) and the stem markers (CD44 and SOX2) in mammary (blue) and NB (green) cell lines. The invasive cell lines (MDA-MB231 and SK-N-BE2) produced the largest spheroids, but each cancer type exhibited differential expressions of stem markers. The MCF-7 mammary cancer cell line only exhibited an increased expression of SOX2, whereas the MDA-MB231 cell line only exhibited an increased expression of CD44. In contrast, the aggressive NB SK-N-BE2 cell line expressed an increase in both markers when cultivated in spheroids.

**Figure 3:**
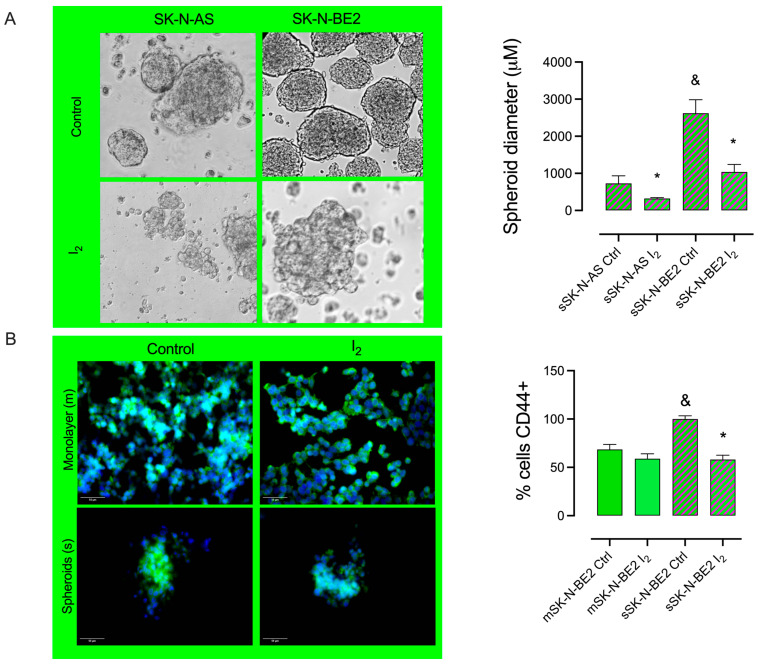
The effect of I_2_ in spheroid formation and CD44 protein content: (**A**) Representative micrograph (10×) of spheroid diameter (μM) and the effect of 200 µM I_2_ in low- (SK-N-AS) and high-grade (SK-N-BE2) NB cell lines. (**B**) The number of positive CD44 cells (immunocytochemistry) in neuroblastoma SK-N-BE2 populations; green fluorescence corresponds to CD44 protein, and blue shows nuclei staining with DAPI. Data represent three independent experiments conducted in duplicate and are expressed as the mean ± SD. Statistical difference (*p* < 0.05) intragroup (*, Student’s *t*-test) and intergroup (&, one-way ANOVA/Tukey’s), respectively.

The comparison of the enrichment of Stem (spheroid formation) in NB cells, summarized in Figure 3A, showed that SK-N-BE2 generates the biggest spheroids, and the presence of I_2_ by 120 h impaired spheroid formation in both cell lines. These results agreed with the diminished protein content of CD44 (immunocytochemistry) in spheroid (s) cultures of SK-N-BE2, supporting that the Stem population expresses higher levels of CD44 protein (Figure 3B).

### 2.3. Induction of Apoptotic and Differentiation Markers in Response to the I_2_ Supplement in SK-N-BE2 Populations

**Figure 4:**
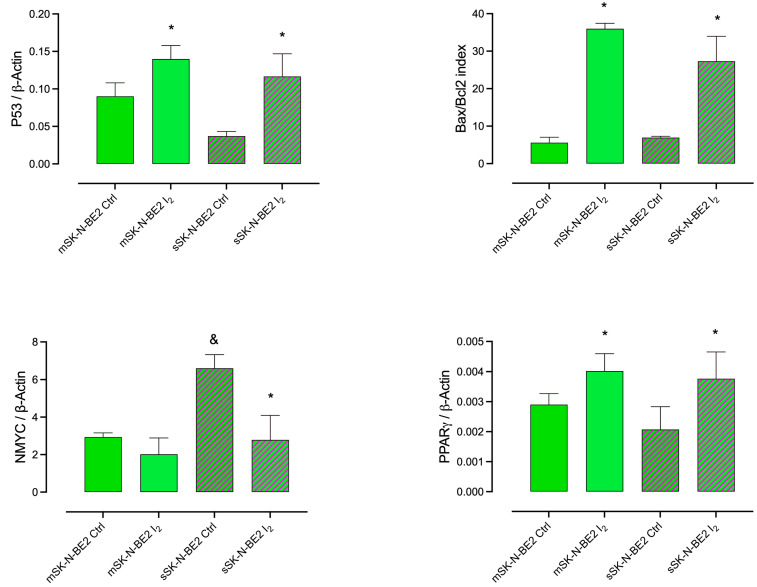
Apoptosis and differentiation responses. Effect of the 200 µM I_2_ supplement on the expression of genes associated with apoptosis (p53, and Bax/Bcl-2 index), Stem (NMYC), and differentiation (PPARγ) in the SK-N-BE2 cell line in monolayer (m) and spheroid (s) cultures. All experiments were carried out for 120 h. Data represent three independent experiments conducted in duplicate and are expressed as the mean ± SD. Statistical difference (*p* < 0.05) intragroup (*, Student’s *t*-test) and intergroup (&, one-way ANOVA/Tukey’s), respectively.

Figure 4 illustrates the effect of the I_2_ supplement on gene expression in SK-N-BE2 NB cell lines cultured as monolayers (m) and spheroids (s). The I_2_ supplement increased the expression of genes associated with apoptosis (p53 and Bax/Bcl-2 index) and differentiation (PPARγ) in both populations. In contrast, I_2_ only reduced the overexpression of NMYC in the Stem population.

### 2.4. The I_2_ Supplement Decreases the Oxidative Mitochondrial Status

**Figure 5:**
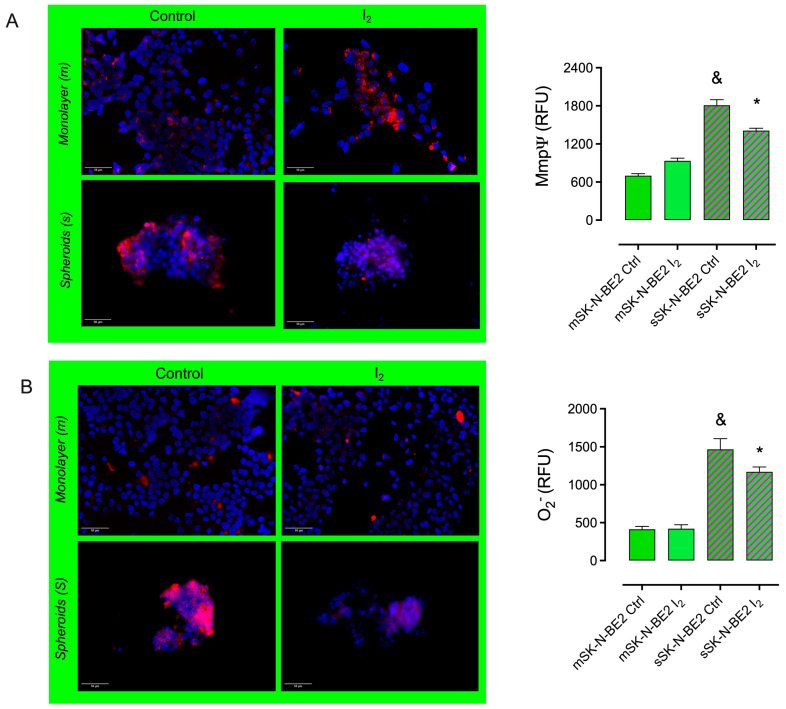
Mitochondrial status. The cell population was supplemented with 200 µM I_2_ for 120 h. Fluorescence assays (RFU: Relative Fluorescence Units) were used to determine membrane permeability: (**A**) MitoTracker entrance, MmpΨ; (10×) and (**B**) superoxide (O_2_^−^) anion content (10×). Data represent three independent experiments conducted in duplicate and are expressed as the mean ± SD. Statistical difference (*p* < 0.05) intragroup (*, Student’s *t*-test) and intergroup (&, one-way ANOVA/Tukey’s), respectively.

Figure 5 shows the mitochondrial oxidative status of monolayer (m) and spheroid (s) SK-N-BE2 cultures and their response after a 200 µM I_2_ supplement. Membrane permeability (MitoTracker entrance; MmpΨ) and superoxide (O_2_^−^) anion content were significantly elevated in Stem (s) compared to progenitors (m). The I_2_ supplement reduced both components only in Stem, demonstrating the direct antioxidant effect of I_2_ on redox status.

### 2.5. I_2_ Supplement Induces the Expression of Antioxidant and Autophagic Pathways

**Figure 6:**
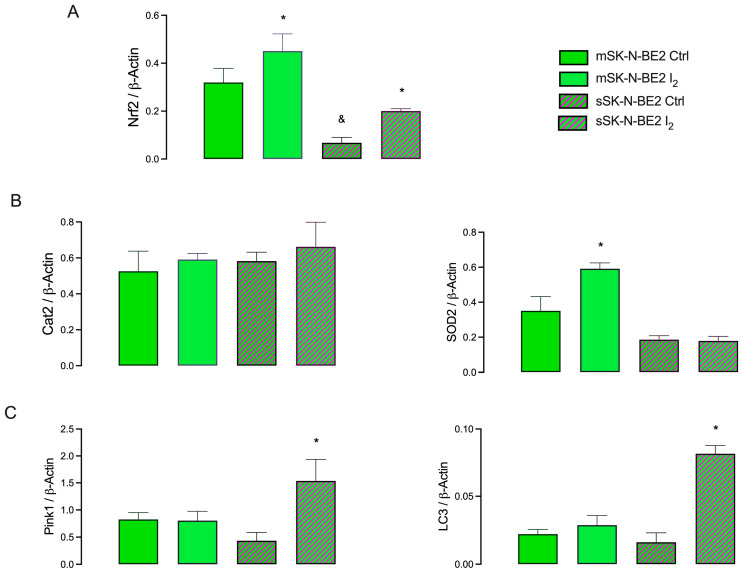
Antioxidant and autophagic responses: (**A**) Expression (RT-qPCR) of the master gene nuclear erythroid factor 2 (Nrf2); (**B**) antioxidants target genes: catalase (Cat) and superoxide dismutase 2 (SOD2); (**C**) autophagy-related target genes: [PTEN-induced putative kinase 1 (Pink1) and microtubule-associated protein 1-light chain3 alpha (LC3)]. Data present three independent experiments conducted in triplicate in monolayer (m) and spheroid (s) cultures and are expressed as the mean ± SD. Statistical difference (*p* < 0.05) intragroup (*, Student’s *t*-test) and intergroup (&, one-way ANOVA/Tukey’s), respectively.

**Figure 7:**
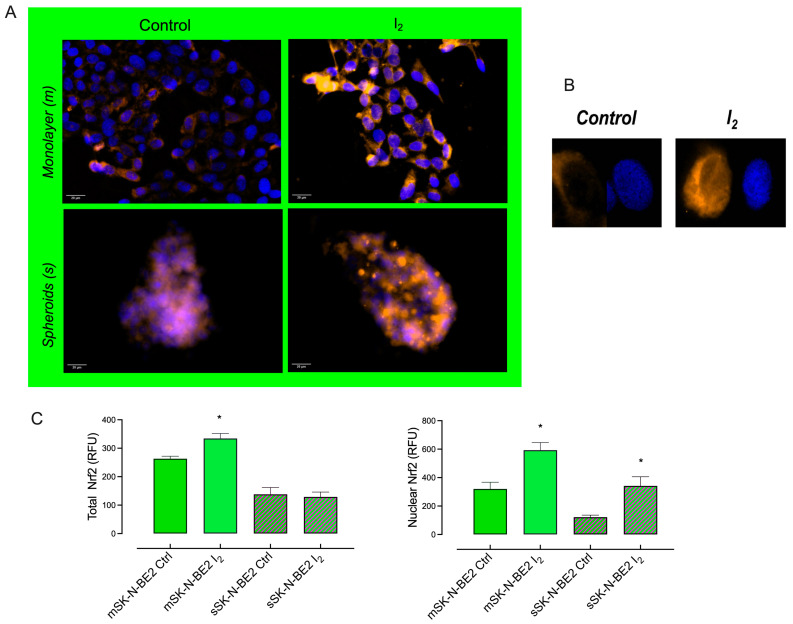
Nrf2 content: (**A**) Representative micrograph (40×) of the number of positive Nrf2 cells (immunocytochemistry) in neuroblastoma SK-N-BE2 populations; yellow fluorescence corresponds to Nrf2 protein, and blue shows nuclei staining with DAPI. (**B**) Micrograph (60×) of control and I_2_ treated cells. (**C**) Total and nuclear quantification of Nrf2 content. Data present three independent experiments conducted in triplicate in monolayer (m) and spheroid (s) cultures and are expressed as the mean ± SD. * indicates statistical differences between groups (Student’s *t*-test; *p* < 0.05).

The nuclear erythroid factor 2 (Nrf2) pathway was analyzed to evaluate the mechanisms associated with the antioxidant and autophagic effects of I_2_. Figure 6 shows that population growth in monolayer (m) cultures exhibited the highest expression of Nrf2 and antioxidant enzymes compared with the Stem population (s). The I_2_ supplement activated the antioxidant pathway [Nrf2, superoxide dismutase 2 (SOD2)] in progenitor populations. In contrast, it activated the autophagic pathway [Nrf2, PTEN-induced putative kinase 1 (Pink1), and microtubule-associated protein 1 light chain3 alpha (LC3)] in Stem populations. The activation of the master gene Nrf2 by I_2_ is corroborated by its translocation into the nucleus in both types of cells, as shown in Figure 7.

### 2.6. I_2_ Supplementation Decreases Invasive Potential Markers in the Stem Population

**Figure 8:**
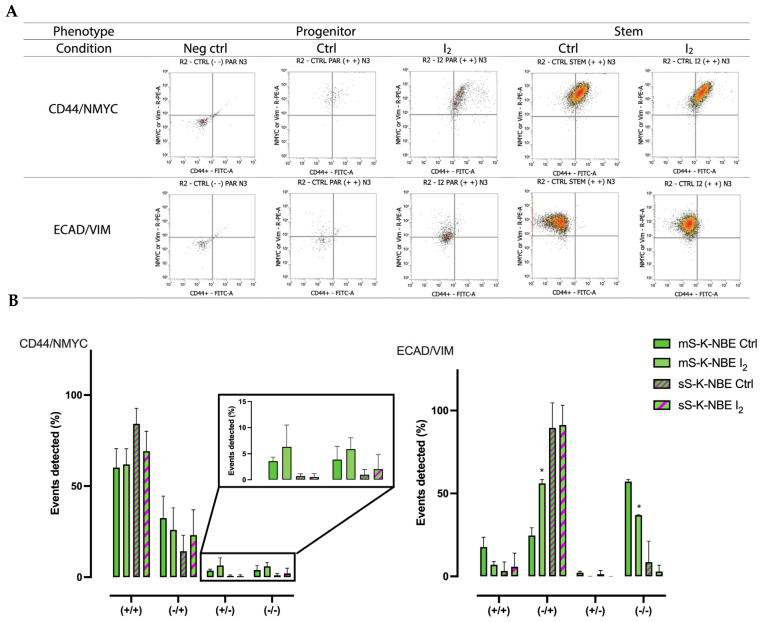
Flow cytometry analysis: (**A**) Representative dotplots of flow cytometry analysis for the different conditions. Progenitor group: negative control group (Neg Ctrl) and (Ctrl and I_2_); Stem group (Ctrl and I_2_). (**B**) Percentage of events detected for the markers CD44/NMYC and ECAD/VIM in different populations: double positive (+/+), single positive (−/+ and +/−), and double negative (−/−). Data represent the mean ± SD of three independent experiments. * Indicates statistically significant differences between groups (*p* < 0.05).

The progenitor and stem populations of the SK-N-BE2 cell line were analyzed by flow cytometry using characteristic markers of stem cells (CD44/NMYC) and invasive potential (E-cadherin/vimentin; ECAD/VIM). Total dot plot flow cytometry for the different conditions is summarized in Figure S1. Figure 8 shows that the CD44+/NMYC+ population is the most abundant in both conditions, corroborating the high aggressiveness of this cell line. I_2_ supplementation did not significantly alter the composition, although it did show a moderate exchange between the decrease in the double-positive subpopulation (CD44+/NMYC+) and the increase in the double-negative subpopulation (CD44-/NMYC-). The cells with the most abundant subpopulation of invasive markers corresponded to ECAD-/VIM+. This profile is considered to have the highest invasive potential. I_2_ supplementation showed a significant decrease in the double-negative subpopulations in both conditions.

### 2.7. I_2_-Preselected SK-N-BE2 Populations Exhibited Minor Capacities of Implantation and Invasion in the In Vivo Zebrafish Xenograft Model

**Figure 9:**
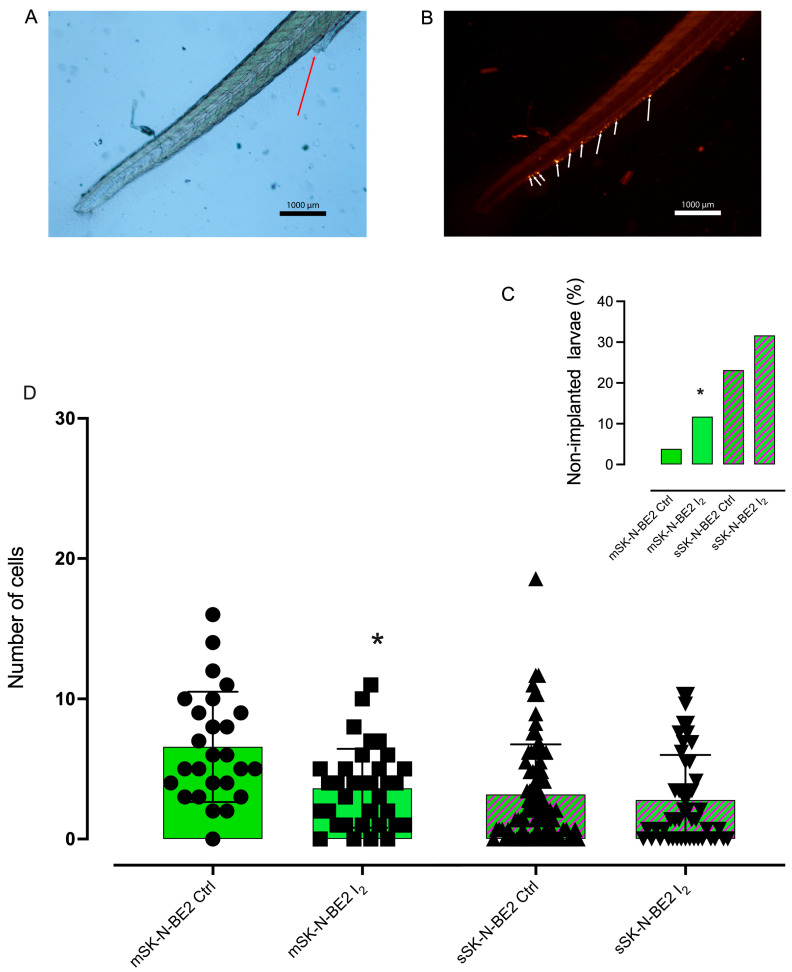
Implantation and invasive capacity in the zebrafish model: (**A**) Representative image of the caudal region of the zebrafish larvae (light field), where the red arrow indicates the larvae anus (4×) (**B**) Representative image of the labeled cells (red, confocal fluorescence), where the white arrows show tumor cells stained with Fast Dil fluorochrome. (**C**) Percentage of larvae that did not show xenografted cells (% of non-implanted larvae) after 96 h. (**D**) Number of cells detected in the larva’s tail after 96 h. Each point represents an individual larva. Graphs represent the mean ± SD. * indicates statistical differences between groups (Student’s *t*-test; *p* < 0.05).

We utilized the in vivo zebrafish xenograft model to investigate the effects of I_2_ on the implantation and invasive capacities of the NB cell population. As shown in Figure 9 fewer Stems were successfully implanted; however, the surviving cells demonstrated a migration capacity to the tail similar to that of the progenitor population. I_2_ administration 96 h before injection significantly reduced the implantation and migration capacities of both populations. A Fisher’s exact test confirmed a significant reduction in the implantation capacity of the progenitor population treated with I_2_ compared to the control (*p* = 0.0183). The odds ratio (OR) was 0.2532 (95% CI: 0.09355 to 0.7916), indicating that cells exposed to I_2_ had approximately 75% lower odds of implantation than the control. The OR reciprocal (3.949, 95% CI: 1.263 to 10.69) suggests that control cells were nearly four times more likely to implant than treated cells.

## 3. Discussion

The importance of studying CSCs lies in the evidence that they seem to be responsible for tumor initiation, therapy resistance, and recurrence [1,6]. This study used spheroids enriched on low-adherence plates without differentiating factors to obtain CSC populations with low (MCF-7 and SK-NAS) and high invasiveness (MDA-MB231 and SK-N-BE2) and compared their sensitivity to the I_2_ supplement. Our results confirm that the most aggressive cell lines exhibit rapid proliferation and greater efficiency in spheroid formation, suggesting a significant presence of the CSC population (Stem). These Stem exhibited overexpression of the CSC markers SOX2, CD44, and NMYC, along with exacerbated mitochondrial membrane permeability.

MCF-7 is classified as a luminal A, low-grade cancer type, characterized by its responsiveness to estrogen and its well-differentiated nature. It exclusively expresses SOX2, which is a basal stem marker. In contrast, MDA-MB231 is a triple-negative, aggressive, and invasive cell line that primarily expresses CD44, with its levels increasing in the Stem population. In NB cells, only the most aggressive and NMYC-positive type (SK-N-BE2) expressed both markers, and again, the Stem population exhibited the highest expression of CD44. The hyaluronan receptor CD44 is a multi-structural and multifunctional cell adhesion molecule involved in cell–cell and cell-extracellular matrix interactions. It acts as a bioactive signaling transmitter engaged in various cellular responses, including inflammation, tumorigenesis, angiogenesis, and metastasis. Also, the overexpression of CD44 often exposes the tumor microenvironment to high levels of reactive oxygen species (ROS) [19]. The treatment with I_2_ showed that the reduction in viability across all populations was correlated with molecular and metabolic changes. The presence of I_2_ significantly decreased CD44 expression in the Stem of SK-N-BE2. This finding is consistent with previous data showing that I_2_ reduces stemness markers (Oct4, Sox2, Nanog, and KLF4) in spheroids from cervical stem cells [15].

At the molecular level, I_2_ treatment modulates master genes in SK-N-BE2, increasing PPARγ, p53, and the Bax/Bcl-2 index in both populations while decreasing NMYC overexpression in Stem. NMYC is a master gene that maintains stem conditions, impairs apoptosis, and promotes resistance [4]. Previous research has shown that I_2_ bound to arachidonic acid generates an iodolipid known as 6-iodolactone (6-IL), which is a specific activator of PPARγ [13]. Treatment with the agonist Rosiglitazone or I_2_ reduced the viability and NMYC expression of SK-N-BE2 cells, and the antagonist GW9662 abolished both effects [17,20]. Also, 15-deoxy-Δ12,14-prostaglandin J2 (15-deoxy-PGJ2), a high-affinity natural ligand of PPARγ, induces the differentiation of L-AN5 NB cells, inhibiting proliferation and neurite outgrowth and reducing NMYC expression [21]. These data support the antineoplastic effect of PPARγ in NB [22] and other cancer types [23].

In relation to oxidative status, it is well known that, like normal stem cells, a low level of ROS has been observed in CSCs in tumors [1]. Changes in ROS levels are crucial to induce the reversible transition between epithelial and mesenchymal states. Active mitochondria exhibiting high ROS generation can promote the transition of quiescent mesenchymal-like state cells to a proliferative epithelial-like state [24]. Our results indicate that enriching spheroids on low-adherence culture plates without differentiating factors may condition a reactivation of high redox status in CSCs, thereby reactivating their proliferation and metastatic potential. Several groups have suggested that I_2_ can act as an oxidant agent in cancer cells by depleting thiol generation and disrupting Mmpψ, which decreases CSC permeability and triggers apoptosis [9]. In this work, we show that Stem exhibited high permeability and superoxide (O_2_^−^) anion content, and the I_2_ supplement significantly decreased these parameters. In addition, our study demonstrates that the oxidant/antioxidant mechanism of I_2_ seems to involve multiple actions, such as increasing the expression of antioxidant enzymes. Greenwald et al. [25] demonstrated a direct interaction between iodine and the Nrf2 pathway through Keap1 iodination, which allows the release and translocation of Nrf2 to the nucleus. Nrf2 is a transcription factor that promotes the antioxidant response to endogenous and exogenous stressors that trigger the expression of phase II protective antioxidant enzymes, such as catalase (Cat) and SOD2 [26]. According to our results, both populations exhibited a significant increase in Nrf2, but only the progenitor population showed a rise in SOD2. In contrast, the increase in Nrf2 expression in Stem was accompanied by the induction of two autophagy-related messengers: PTEN-induced kinase 1 (Pink1) and the microtubule-associated protein1 light chain3 alpha (LC3). It has been previously described that, depending on the cellular context, the master gene Nrf2 may serve as an essential factor for cell survival by inducing mitophagy via the activation of the Pink1/parkin pathway [26]. Pink1 is a kinase that functions as a mitochondrial damage sensor that recruits the cytosolic E3 ubiquitin ligase Parkin from the cytosol to the outer mitochondrial membrane and promotes the degradation of ubiquitinated proteins. With the help of LC3-II, ubiquitinated proteins direct the damaged mitochondria toward the lysosome for degradation by proteasomal enzymes [27]. However, depending on the context, mitophagy in cancer can function as either a tumor suppressor or a tumor promoter. As a tumor suppressor, it preserves cellular integrity but impairs the evolution toward a more aggressive, invasive, or chemoresistant (decreased chromosomal instability) cell type. As a pro-oncogenic agent, it maintains the survival of cancer cells by inhibiting cell apoptosis after antitumor therapy. Although the cancer cell survives in both conditions, the cellular context will generate a vulnerable tumor cell lacking metastatic capacity in the first case or a chemoresistant cell in the second case [24]. In our work, I_2_ supplementation in Stem increased both apoptosis and mitophagy messengers, resulting in loss of viability and spheroid formation. These conditions have been described previously in the presence of antioxidants [28,29].

Finally, our results showed that I_2_ supplementation generated a selection of populations within cell cultures that altered their carcinogenic capabilities. Flow cytometry analysis confirmed that the most abundant subpopulations in the SK-N-BE2 cell line exhibited characteristics of an aggressive phenotype: chemoresistance (CD44+/NMYC+) and invasive potential (ECAD^−^/VIM^+^). I_2_ slightly decreased these populations but significantly modified their implantation and migration capabilities in the in vivo model. Indeed, the few changes observed in the Stem population in the presence of I_2_ may support the hypothesis that mitophagy induction reduces the adaptability of this cancer cell, thereby preventing its implantation in the zebrafish microenvironment [30]. Research is currently underway to identify additional molecular changes associated with these effects.

## 4. Materials and Methods

### 4.1. Chemicals and Reagents

MammoCult™ Human Basal Medium, Mammocult™ Proliferation Supplement, and Hydrocortisone were obtained from Stemcell Technologies (Vancouver, BC, Canada). Heparin sodium salt was acquired from Sigma-Aldrich (St. Louis, MO, USA) and the basement membrane matrix Matrigel from Corning (Bedford, MA, USA). Dulbecco’s Modified Eagle’s Medium (DMEM) and fetal bovine serum (FBS) were from BIOWEST (Nuaillé, France). Penicillin–streptomycin and trypsin–EDTA solutions were provided by GIBCO (Grand Island, NY, USA). Sublimed iodine was obtained from Macron-Avantor (Center Valley, PA, USA). The iodine solutions were titrated with sodium thiosulfate. All other reagents were of the highest purity grade available.

### 4.2. In Vitro

#### 4.2.1. Cell Culture

The MCF-7 (HTB-22) and MDA-MB-231 (CRM-HTB-26) breast cancer cell lines and SK-N-AS (CRL-2137) and SK-N-BE2 (CRL-2271) NB cell lines were purchased from the American Type Culture Collection (ATCC, Manassas, VA, USA). The assays were performed at 5–10 passages, and cell lines were authenticated by STR profiling (BIMODI Invoice number 190320-029). Monolayer (m) cell culture was grown in DMEM supplemented with FBS (10%) and penicillin/streptomycin (2%) from Invitrogen (Waltham, CA, USA) in a humidified chamber with a 5% CO_2_ atmosphere at 37 °C.

#### 4.2.2. Spheroids (Stem)

To develop tumor spheroids derived from the cancer cell lines, monolayer cell cultures were grown in adherent cell culture plates and then harvested 24 h later, washed with phosphate buffer solution (PBS), trypsinized, and counted. A total of 1,500,000 cells were seeded in non-adherent culture plates with MammoCult™ Human Basal Medium (Stem Cell Technologies, Vancouver, BC, Canada), supplemented with 10% MammoCult™ Proliferation Supplement, 0.48 µg/mL Hydrocortisone, 0.04% heparin solution, and 4 µg/mL and 100 U/mL penicillin/streptomycin. Cells were cultured at 37 °C in a humidified atmosphere with 5% CO_2_ for 96 or 120 h, and spheroid formation was monitored daily.

#### 4.2.3. Cell Proliferation

After 120 h of differentiation, the monolayer (m) or spheroid (s) cultures of breast cancer and NB cells were treated with 200 μM I_2_ for 96 h. The I_2_ was previously diluted in the culture medium (DMEM or MammoCult) and then added to the cells. After treatments, cultures were detached and mixed with trypan blue dye (0.04%) for cell counting with a hemocytometer; viability is reported as fold change against control. Three independent experiments were conducted in duplicate.

#### 4.2.4. Immunocytochemistry

Monolayer and spheroid cultures were washed with PBS, then fixed with paraformaldehyde and labeled with the stemness marker using the membrane protein antibody anti-human-CD44/FITC (ab182981; 1:2500, Abcam, Cambridge, UK) or anti-human-Nrf2 (sc-365949; 1:100, Santa Cruz Biotechnology, Dallas, TX, USA) with Alexa Fluor 546 (A-11081; 1:1000, Thermo Fisher, Waltham, MA, USA) according to the manufacturer’s recommended procedures. The glass slides were mounted with anti-FADE and 2-(4-amidinophenyl)-1H 1H-indole-6-carboxamidine (DAPI). The percentage of cells positive for CD44 was quantified with ImageJ 1.8 software (National Institutes of Health, Bethesda, MD, USA) by randomly analyzing 100 cells per slide (n = 4).

#### 4.2.5. Mitochondrial Function

Monolayer and spheroid cultures were treated for 48 h with 200 μM of I_2_. After treatments, the cells were washed with PBS and labeled with 200 nM MitoTracker Red CM-H2Xros (Thermo Fisher, Waltham, MA, USA) or MitoSOX Red (Thermo Fisher, Waltham, MA, USA) and incubated at 37 °C for 45 min. The cells were fixed for 10 min with cold ethanol, PBS-washed, mounted with anti-FADE and DAPI, and mounted with Stellan. The mitochondrial functional state was evaluated at micrographs taken by an epifluorescence microscope (Axio Imager, Carl Zeiss, Jena, Germany) and analyzed with ImageJ 1.8 software (National Institutes of Health, Bethesda, MD, USA) to quantify the relative fluorescence units as previously described [14].

#### 4.2.6. Transcriptional Response

SOX2, CD44, P53, Bax, Bcl-2, Nrf2, Cat, SOD2, Pink1, LC3, and PPARγ were analyzed by RT-qPCR from cell monolayer and spheroid cultures. Briefly, the RNA was obtained using Trizol reagent (Life Technologies, Inc., Carlsbad, CA, USA). Two micrograms of RNA were reverse transcribed (RT) using oligo-deoxythymidine (Invitrogen, Waltham, MA, USA). Real-time PCR (qPCR) was performed using the SYBR Green amplification marker. The Rotor-Gene 3000 sequence detector system (Corbett Research, Mortlake, NSW, Australia) was used with SYBR Green as a DNA amplification marker (gene-specific primers are listed in Table S1). Relative mRNA levels were normalized to the mRNA level of β-actin.

#### 4.2.7. Flow Cytometry

Flow cytometry was performed using the Attune^®^ NXT acoustic focusing cytometer (Thermo Fisher Scientific, Carlsbad, CA, USA) to quantify stemness-related proteins. Parental cells were trypsinized, washed with PBS, and centrifuged, while spheroids were collected, washed, and dissociated after fixation. All samples were fixed in pre-chilled (−20 °C) 90% ethanol and stored at −20 °C. For staining, cells and spheroids were incubated with fluorochrome-conjugated antibodies (summarized in Table S2) in PBS at 4 °C for 30 min, washed, and filtered through a 70 µm nylon strainer before cytometric analysis. A minimum of 10,000 events in the cell gate were collected for each sample to analyze the individual event packages. A dotplot was generated by comparing forward scatter-A against forward scatter-H, and any doublets were excluded. Dotplots of flow cytometry analysis for the different conditions are summarized in Figure S1.

### 4.3. In Vivo

#### 4.3.1. Zebrafish Xenograft Model

Larval zebrafish (*Danio rerio*) were provided by the zebrafish laboratory at the vivarium of the Institute of Neurobiology, UNAM (INB-UNAM). All procedures conducted by the Animal Care and Use Program (National Institutes of Health, USA) were approved by the Research Ethics Committee (Protocol #124.A) of the INB-UNAM. Larvae were kept at 28 °C in E3 embryo medium, which contained per liter 0.286 g NaCl, 0.0126 g KCl, 0.048 g CaCl_2_·2H_2_O, and 0.081 g MgSO_4_·7H_2_O, with a pH of 7.2 and supplemented with 0.2 mM 1-phenyl-2-thiourea (PTU). PTU was added at 24 h post-fertilization to inhibit pigmentation. SK-N-BE2 populations were labeled with 4 mg/mL PBS Fast Dil dye™ (Invitrogen). Cells were resuspended in a PBS injection medium at 200,000 cells/µL and manually loaded into borosilicate glass capillary needles (1 mm O.D.: 90.78 mm I.D.; #30-0035, Harvard Apparatus, Holliston, MA, USA). Larvae were anesthetized using a 0.08 mg/mL tricaine solution (Sigma-Aldrich), placed on 1.5% agarose, and injected using a microinjector (Eppendorf, FemtoJet 49, Hamburg, Germany). Approximately 500 Dil-labeled cells were injected into the perivitelline space of two-day-old zebrafish larvae. The injection parameters were 80–200 hPa pressure and 0.1–0.3 s time. Larvae were incubated at 28 °C in E3/PTU medium, and cancer cell dissemination to the caudal vein plexus was analyzed with a fluorescence Nikon Eclipse E-600 microscope (Tokyo, Japan) at 10×/0.30 magnification after 72 h. Larvae were anesthetized with tricaine solution (Sigma-Aldrich), imaged using NIS-Elements Imaging software (v5.30.04 64 bits) and euthanized by tricaine overdose and a freezing method [27]. Images were analyzed with ImageJ. Experiments were conducted under random conditions. At least 25 larvae were injected per condition, and the experiment was performed on two separate days.

#### 4.3.2. Statistical Analysis

Molecular analysis used two replicate wells per group, and the experiment was performed in triplicate. A one-way ANOVA followed by Tukey’s test was used for post hoc analysis between groups (*p* < 0.05). In vivo analysis included at least 25 larvae injected per condition, and the experiment was conducted on two separate days. A Student’s *t*-test was used to compare control and I_2_ conditions for each population (*p* < 0.05). Additionally, Fisher’s exact test was used to evaluate implantation capacity, and OR analysis was performed to quantify the effect of I_2_ treatment on implantation probability.

## 5. Conclusions

These data indicate that I_2_ interferes with viability, implantation, and invasion capacity in all cell lines, but the molecular mechanisms appear to be different depending on the progenitor or Stem condition.

## Data Availability

The data presented in this study are available upon specific request from the corresponding author.

## Supplementary Materials

The following supporting information can be downloaded at https://www.mdpi.com/article/10.3390/ijms26094020/s1.
