# Supplementary material for: Molecular Iodine Exhibited Differential Antiproliferative Actions in Progenitor and Stem Populations from Chemoresistant Cancer Cells
Source: Int J Mol Sci. 2025 Apr 24;26(9):4020. doi: 10.3390/ijms26094020 (PMC12072113; doi:10.3390/ijms26094020)

Supplementary material

Table. S1. Oligos sequences used to qPCR

Gen	Reference	Sense	Antisense	bp	Ta (°C)
**SOX2**	NM_003106.4	CACCTACAGCATGTCCTACTC	CATGCTGTTTCTTACTCTCCTC	386	58
**CD44**	NM_000610.4	AGAAGGTGTGGGCAGAAGAA	AAATGCACCATTTCCTGAGA	116	60
**PPARγ**	NM_138711.6	CGACATTCAATTGCCATGAG	GACCACTCCCACTCCTTTGA	257	58
**NMYC**	NM_001293228.2	ACCCTGAGCGATTCAGATGAT	GTGGTGACAGCCTTGGTGTT	113	62
**Nrf2**	NM_006164.5	CAGTCAGCGACGGAAAGAGT	AGTGACTGAAACGTAGCCGA	312	62
**CAT**	NM_001752.4	TCCGGGATCTTTTTAACGCCATTG	TCGAGCACGGTAGGGACAGTTCAC	362	62
**SOD2**	NM_000636.4	GGCCTACGTGAACAACCTGA	CACGTTTGATGGCTTCCAGC	203	62
**Pink1**	NM_032409.3	GGACACGAGACGCTTGCA	TTACCAATGGACTGCCCTATCA	63	62
**LC3**	NM_001085481.3	GAGAAGCAGCTTCCTGTTCTGG	GTGTCCGTTCACCAACAGGAAG	138	62
**P53**	NM_001126118.2	CCATGAGCGCTGCTCAGATA	GGGCACCACCACACTATGTC	124	60
**Bax**	NM_138764.5	AAGCTGAGCGAGTGTCTCAAGCGC	TCCCGCCACAAAGATGGTCACG	327	60
**Bcl-2**	NM_000633.3	CTCGTCGCTACCGTCGTGACTTCG	CAGATGCCGGTTCAGGTACTCAGTC	242	60
**β-Actin**	NM_001101.5	CCATCATGAAGTGTGACGTTG	ACAGAGTACTTGCGCTCAGGA	175	58

Table S2. Antibodies used in cytometry analysis

Target	Conjugate	Manufacturer (Cat. No.)	Dilutions
**CD44**	FITC	BD Pharmingen (560977)	1:25
**VIM**	PE	BD Pharmingen (562337)	1:25
**ECAD**	Alexa Fluor ™ 647	BD Pharmingen (560062)	1:25
**NMYC**	PE	NOVUS Biologicals (NB200-109PE)	1:25

Figure S1. Flow cytometry dotplots.

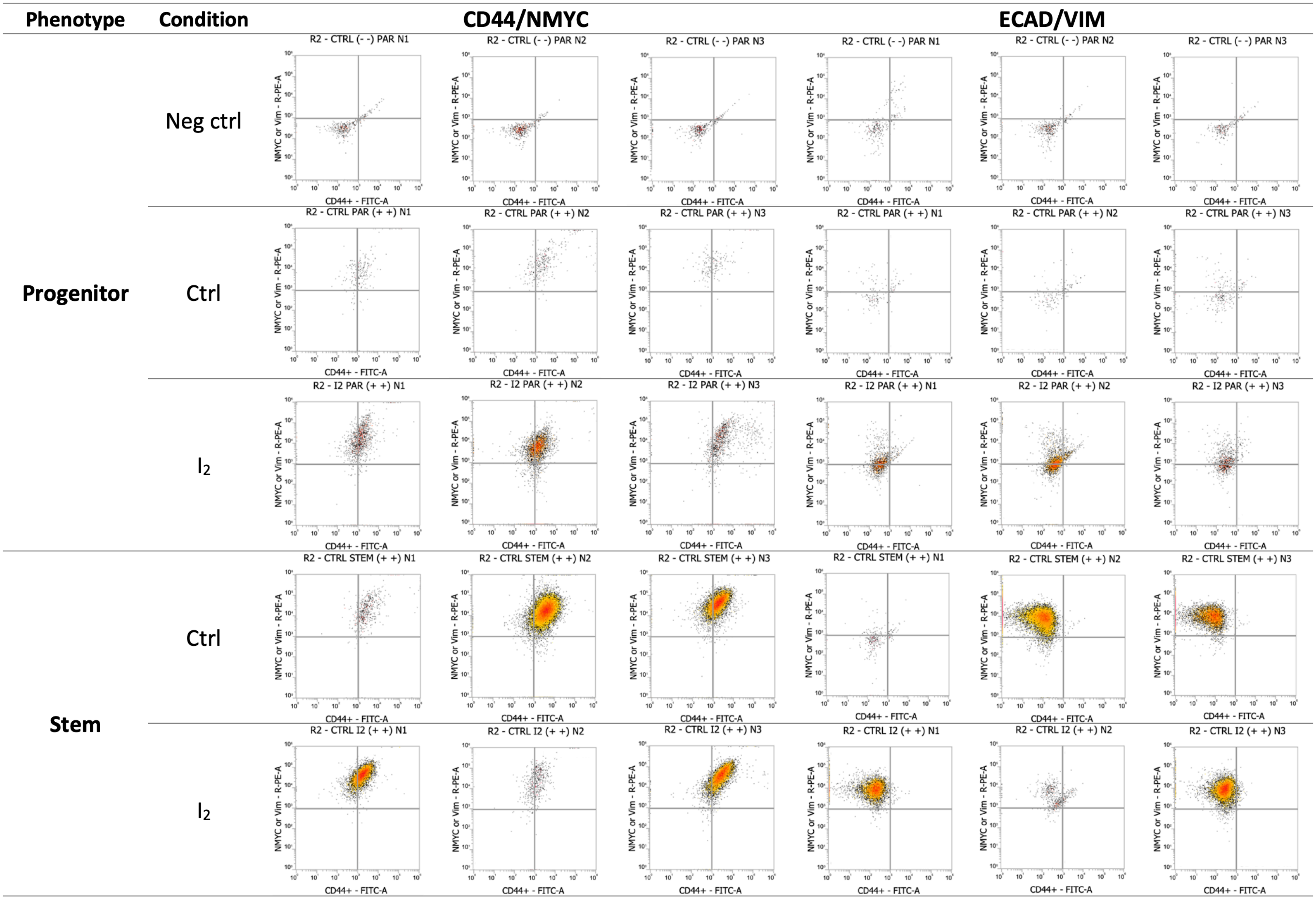

Supplement: Supplementary file 1 [file ijms-26-04020-s001.zip › ijms-3562466-supplementary.pdf]
